# High-Frequency Skywave Source Geolocation Using Deep Learning-Based TDOA Estimation and Bias-Regularized Semidefinite Programming with Field Evaluation

**DOI:** 10.3390/s26092755

**Published:** 2026-04-29

**Authors:** Chen Xu, Houlong Ai, Le He, Chaoyu Hu, Siyi Chen, Zhaoyang Li, Xijun Liu

**Affiliations:** College of Aviation Electronic and Electrical Engineering, Civil Aviation Flight University of China, Chengdu 641400, China; xuchen@cafuc.edu.cn (C.X.); ahlcafuc@126.com (H.A.); hlcafuc@126.com (L.H.); hcycafuc@126.com (C.H.); sychen@cafuc.edu.cn (S.C.); lzy2024@cafuc.edu.cn (Z.L.)

**Keywords:** HF skywave geolocation, TDOA estimation, ionospheric channel modeling, multi-weight GCC, semidefinite relaxation

## Abstract

High-frequency (HF) skywave propagation exploits ionospheric reflection for beyond-line-of-sight transmission, making time-difference-of-arrival (TDOA)-based geolocation a primary technique for localizing non-cooperative HF emitters. However, reliable TDOA estimation remains challenging due to time-varying ionospheric conditions, wideband multipath dispersion, and low signal-to-noise ratio (SNR). This paper proposes an integrated framework coupling realistic channel synthesis, deep learning-based TDOA estimation, and convex optimization-based localization. Three contributions are made. First, an improved wideband ionospheric channel model is constructed by integrating the International Reference Ionosphere (IRI) with region-specific calibration and a stochastic perturbation module, yielding time-varying multipath responses for physics-consistent waveform generation. Second, a convolutional neural network (CNN)-based TDOA estimator is designed to jointly exploit time-domain complex-baseband in-phase/quadrature (I/Q) waveforms, multi-weight generalized cross-correlation (GCC) feature maps, and channel-state information (CSI) within a unified regression network, achieving robust delay estimation under severe noise and multipath conditions. Third, the geolocation problem is formulated as a bias-regularized constrained least-squares problem with unknown ionospheric excess-delay surrogates, and a semidefinite programming (SDP) relaxation is derived to yield a tractable solution without prescribing a fixed virtual reflection height. Simulations show that the proposed estimator consistently outperforms competing algorithms across a wide SNR range and narrows the gap to the Cramér–Rao lower bound (CRLB) at high SNR. On field-recorded signals, the estimator reduces the mean absolute TDOA deviation by 51% relative to GCC with phase transform (GCC-PHAT), and the end-to-end pipeline achieves a mean geolocation error of 19.67 km across 100 field segments, outperforming all compared baselines.

## 1. Introduction

High-frequency (HF) skywave signals achieve long-range propagation via ionospheric reflection and are widely employed for emitter localization in scenarios such as military operations and disaster response. Accurate time-difference-of-arrival (TDOA) estimation is central to these applications, yet remains challenging owing to ionospheric time variability, multipath propagation, and low signal-to-noise ratio (SNR).

In multistation localization based on hyperbolic positioning, estimating the inter-receiver time offset is critical [1,2]. In passive systems, the key measurements are the arrival-time differences between multiple receivers and a designated reference receiver; improved TDOA precision translates directly into higher localization accuracy. This precision depends on both inter-receiver time synchronization and the estimation algorithm employed.

Existing TDOA estimation methods can be broadly classified into three categories. The first category comprises classical correlation-based methods, in which time-difference estimation is achieved by computing the cross-correlation of the received signals [3,4,5]. Among these methods, the generalized cross-correlation (GCC) method, especially the phase-transform variant (GCC-PHAT) [6], remains a widely used engineering solution because of its simplicity and low implementation cost. However, its performance can deteriorate markedly under low-SNR, multipath, and dispersion-dominated propagation conditions. The second category includes improved correlation or time–frequency enhancement methods, such as refined SRP/GCC-PHAT formulations that analyze bandwidth–resolution tradeoffs, time–frequency reassignment or synchrosqueezing methods for noise suppression, and iteratively reweighted SRP-PHAT variants for multi-array or multi-source scenarios [7,8]. Although these methods improve robustness to some extent, they still fundamentally rely on handcrafted correlation structures and often remain sensitive to severe channel distortion and rapidly time-varying propagation effects. The third category is data-driven estimation, which attempts to learn robust delay mappings directly from data [9]. Such methods offer greater flexibility under adverse conditions, but their effectiveness depends strongly on the representativeness of the training data and the realism of the adopted channel model.

During HF skywave propagation the ionosphere exhibits pronounced spatiotemporal heterogeneity. The critical frequencies, electron density profiles, and attenuation coefficients of its layered structure (D, E, and F regions) fluctuate with geographic location, solar activity, and diurnal cycle. These variations manifest as group-delay fluctuations, multipath dispersion, and Doppler shifts, all of which can significantly degrade cross-correlation-based time-difference measurements. In multi-sensor TDOA systems, the communication and storage burden scales as O(M) for a reference-receiver architecture and up to $O(M^{2})$ for full pairwise processing of high-rate in-phase/quadrature (I/Q) data, which can become prohibitive in large deployments. This bottleneck has motivated data-driven regressors for robust delay inference under adverse channel conditions.

In this work, we adopt a reference-receiver formulation. The proposed estimator operates on one receiver pair at a time: each forward pass takes two synchronized receiver streams as input and outputs a single TDOA estimate. For a system with *M* receivers, the full reference-based TDOA set is obtained by fixing one receiver as the reference and applying the estimator to the remaining M−1 pairs, thereby maintaining linear scaling with the number of receivers. In all experiments, GCC-derived correlation features are used as part of the estimator input, and the resulting delay estimates are then passed to the localization stage described in Section 4.

Existing HF skywave localization methods can also be grouped into several representative categories. The first category consists of fixed-height or empirical virtual-reflection-height methods, which approximate the ionospheric path by a simplified geometric model and reduce the problem to two-dimensional hyperbolic positioning. These methods are computationally convenient, but their accuracy degrades when the assumed virtual height deviates from the actual propagation condition. The second category includes ionosphere-model-assisted or ray-tracing-assisted methods, which refine path estimation by incorporating physical ionospheric information. While more realistic, they usually require prior ionospheric knowledge that is difficult to obtain accurately in dynamic environments. The third category comprises iterative nonlinear optimization methods based on TDOA equations. These methods can provide refined solutions, but the underlying formulations are generally nonconvex and may suffer from sensitivity to initialization and convergence to local minima. A fourth category includes convex-relaxation-based approaches for unknown ionospheric environments [10]. In addition, recent studies have explored direct localization and quasi-parabolic ionospheric solvers [11,12], as well as land-based HF geolocation architectures [13]. Although these methods have advanced the field, their robustness under realistic HF channels with strong uncertainty, time variation, and multipath distortion remains limited. This motivates the development of a more integrated framework that simultaneously improves channel realism, TDOA robustness, and end-to-end localization performance.

The authors previously investigated HF skywave geolocation under unknown ionospheric environments via convex relaxation [10] and reported an improved TDOA processing method with field experiments for land-based long-range HF skywave geolocation [14]. The present manuscript differs from [10,14] in both scope and technical focus: it introduces an IRI-augmented stochastic wideband channel model with time-varying disturbances, develops a CNN-based fusion TDOA estimator that jointly exploits raw waveforms, channel descriptors, and multi-weight GCC features, and evaluates how the improved TDOA estimates propagate to end-to-end geolocation under both simulation and field conditions.

The key contributions are summarized as follows:

**(1)**  **Improved Ionospheric Channel Modeling.** We develop an enhanced wideband HF ionospheric channel model based on the classical Institute for Telecommunication Sciences (ITS) structure [15], augmented with International Reference Ionosphere (IRI) parameters, region-specific calibration using Chinese Reference Ionosphere (CRI) coefficients, and a stochastic perturbation module. The model is calibrated for the Chinese sector (15–55° N, 70–140° E) and reproduces key statistical characteristics of time-varying wideband HF multipath, providing a controllable testbed for training and stress-testing the proposed estimator. Regional adaptation may be required for other geographic areas.
**(2)**  **CNN-Based Multi-GCC TDOA Estimation.** We develop a CNN-based TDOA estimator that fuses multiple established GCC weightings within a regression network. Our contribution is to stack complementary correlation views—PHAT, Smoothed Coherence Transform (SCOT), and Maximum-Likelihood (ML) weighting over a fixed lag aperture—encode them with a lightweight CNN branch, and fuse them with raw-waveform features and optional channel-state descriptors in a single end-to-end regressor. This design avoids committing to a single handcrafted weighting and improves robustness to noise and multipath relative to classical single-weight GCC baselines.
**(3)**  **Bias-Regularized LS–SDP Geolocation Framework.** Building on the high-accuracy TDOAs from the CNN, we formulate HF source localization as a bias-regularized constrained least-squares problem in which the source position, the common transmit-time parameter, and receiver-specific nonnegative squared-range correction terms are jointly estimated. A semidefinite programming (SDP) relaxation converts the nonconvex problem into a tractable convex program solvable by interior-point methods, enabling robust geolocation without prior ionospheric reflection heights.

Together, these contributions form an end-to-end pipeline bridging realistic ionospheric modeling, deep learning-based TDOA estimation, and convex optimization for accurate HF skywave source localization under complex and uncertain ionospheric conditions.

The remainder of this paper is organized as follows. Section 2 introduces the improved HF ionospheric channel model. Section 3 presents the CNN-based multi-GCC TDOA estimation method. Section 4 formulates the geolocation problem and derives the SDP relaxation. Section 5 reports simulation and field-experiment results. Section 6 provides concluding remarks and future directions.

## 2. Enhanced HF Ionospheric Channel Model

HF ionospheric channels exhibit pronounced multipath and time-varying characteristics that are often inadequately captured by conventional models. The ITS wideband channel model, proposed by Vogler and Hoffmeyer [15], is a classical framework for HF multipath propagation. It employs a time-varying impulse response h(t,τ) to characterize the ionospheric channel as a function of time *t* and delay τ. Building on the ITS structure, this paper integrates the International Reference Ionosphere (IRI) model and introduces a parameter perturbation module, yielding a wideband HF channel model that captures stochastic and time-varying ionospheric behavior. The proposed architecture comprises three components: (1) an IRI module with regional calibration to generate background ionospheric parameters, (2) a parametric correction module that applies perturbations driven by seasonal and diurnal variations, and (3) an enhanced ITS channel model for multipath and Doppler profile synthesis.

**Figure 1:**
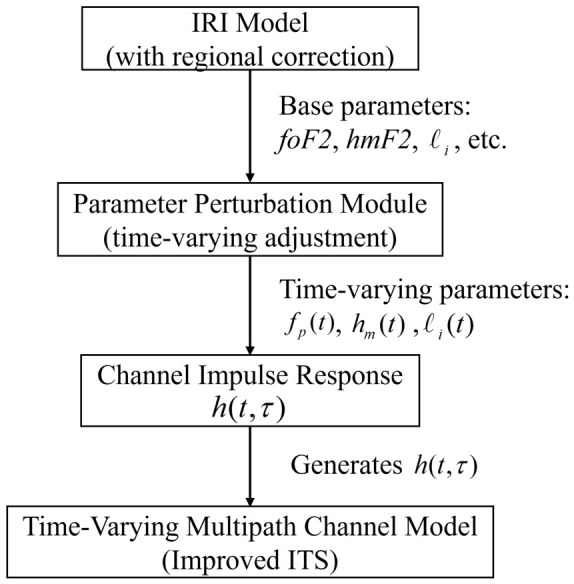
Overall architecture of the improved HF ionospheric channel model.

Figure 1 illustrates the overall architecture. The IRI module generates background ionospheric parameters, which are processed by the perturbation module to produce time-varying realizations. These are fed into the enhanced ITS model to synthesize h(t,τ). The model is validated at the statistical level against published HF sounding measurements (Table 1) and is restricted to the Chinese sector, where regional ionosonde data are available for calibration.

### 2.1. IRI Model with Regional Calibration

The IRI is a global empirical ionospheric model that has been maintained since the 1960s by a joint COSPAR and URSI working group [20]. It provides climatological parameters characterizing the ionosphere’s average behavior under specified temporal, geographic, and solar–geomagnetic conditions, including layer-specific critical frequencies (e.g., $f_{o}F_{2}$) and peak heights. The original ITS model requires inputs such as signal path distance *d*, penetration frequency $f_{p}$, peak electron density height $h_{m}$, and the layer-thickness parameter $l_{i}$ for ionospheric layer *i*—parameters that typically rely on in situ measurements and can introduce systematic bias when uncorrected global-model outputs are used directly. To improve regional accuracy, our model employs a regionally calibrated IRI module. The following enhancements are implemented for the Chinese sector:

- **Regional Coefficient Substitution.** The standard IRI uses CCIR (Comité Consultatif International des Radiocommunications) and URSI (Union Radio-Scientifique Internationale) global coefficients to predict the F2-layer critical frequency $f_{o}F_{2}$ and the propagation factor $M\left(3000\right)F_{2}$. We replace these with the coefficient set from the Chinese Reference Ionosphere (CRI) [21,22,23], a regional empirical model constructed from local ionospheric observations. This substitution is applied only within the Chinese sector and is intended to improve the fidelity of $f_{o}F_{2}$ and $M\left(3000\right)F_{2}$ used to drive channel synthesis.
- **Peak Height Calibration.** Using the derived $f_{o}F_{2}$ and $M\left(3000\right)F_{2}$, the IRI’s embedded formulas compute the peak heights of the E layer ($h_{mE}$), F1 layer ($h_{mF1}$), and F2 layer ($h_{mF2}$). These are compared against regional ionosonde measurements; if deviations exceed 5%, we iteratively adjust the height-formula coefficients until relative errors fall below this threshold (e.g., after calibration, $h_{mE}\approx 115$ km for the Chinese sector). This calibration reduces systematic bias in the background layer-height parameters but is not intended to reproduce event-level ionospheric variability for a specific link and time. Accordingly, the synthesized channels should be interpreted as statistically plausible realizations conditioned on month and local time, not as replays of particular measured instances. We note that the CRI-based substitution and peak-height calibration are region-specific; when the framework is applied elsewhere or under extreme ionospheric conditions, recalibration using local observations is recommended.

After these corrections, the IRI module outputs key background parameters for each reflection layer: critical frequencies (e.g., $f_{o}F_{2}$), peak electron density heights (e.g., $h_{mF2}$), and layer-thickness parameters $l_{i}$. Once peak heights are determined, the local electron density distribution can be reconstructed via a multi-layer quasi-parabolic segment (QPS) model and combined with the Appleton–Hartree equation to obtain spatial refractive-index distributions for ray tracing and channel-characteristic analysis.

### 2.2. Parameter Perturbation Module

The ionosphere is not a stable homogeneous medium but a random-parameter channel with spatiotemporal fluctuations. After obtaining the mean background from the calibrated IRI module, a perturbation module is incorporated to emulate ionospheric nonstationarity by introducing time-varying stochastic factors into the critical propagation parameters.

Ionospheric parameters exhibit superimposed periodic and stochastic variations across diurnal and seasonal cycles. The proposed model categorizes scenarios by reflection layer (E, F1, or F2), month, local time, and simulation timeline. For each scenario, the perturbation is decomposed into two terms:

- F(month,t): a deterministic trend function (units matching the ionospheric parameter $X_{i}$, e.g., MHz or km) that captures seasonal and diurnal patterns, such as solar-driven ionization increases in $f_{o}F_{2}$ during daylight and electron density decay at night.
- G(month,t,Δt): a stochastic fluctuation term (same units) that injects short-term variability. To capture temporal correlation, *G* is modeled as a first-order correlated Gaussian process: $G\left[k+1\right]=\rho_{G}\;G\left[k\right]+\eta \left[k\right],\;\eta \left[k\right]∼N\;\left(0,\;\sigma_{\eta }^{2}\left(\mathrm{month},t\right)\right),$ where $\rho_{G}\in \left(0,1\right)$ controls the correlation time and $\sigma_{\eta }\left(\mathrm{month},t\right)$ controls the fluctuation amplitude. We set Δt=60 s and $\tau_{c}=10$ min, yielding $\rho_{G}=\exp\left(-\Delta t/\tau_{c}\right)\approx 0.905$, consistent with reported ionospheric parameter correlation times of 8–15 min in mid-latitude HF sounding campaigns [16,18]. The innovation amplitude is $\sigma_{\eta }=\alpha \;{\bar{X}}_{i}$ with α=0.07, matching the 5–10% relative variability of the F2-layer critical frequency reported in [16,20].

For each reflection layer i∈{E,F1,F2}, let ${\bar{X}}_{i}$ denote the background baseline value from the calibrated IRI module. The time-varying realization at the next step is

(1) $$X_{i}\left(t+\Delta t\right)={\bar{X}}_{i}+F_{i}\left(\mathrm{month},t\right)+G_{i}\left(\mathrm{month},t,\Delta t\right),$$

ensuring that parameters such as $f_{p}$, $h_{m}$, and the layer-thickness parameter $l_{i}$ exhibit gradual temporal variations rather than remaining constant. During day–night transitions, an exponentially decaying term may be superimposed to emulate the rapid decline in F-region electron concentration, while a smooth sinusoidal recovery term may be added after sunrise. Such non-uniform perturbation functions better reproduce unstable ionospheric structures during these transitions.

In implementation, *F* and *G* are precomputed from statistical and theoretical analyses and stored in a lookup table; during simulation they are retrieved based on the current month and local time. As reflected by the measured-data results in Section 5, residual simulation-to-measurement mismatch can remain, and the proposed estimator is therefore evaluated on both synthetic and field datasets.

### 2.3. Improved ITS Multipath Time-Varying Channel Model

In the original ITS model, the impulse response is formed by superposing multiple independent propagation paths, each corresponding to a distinct mode (e.g., high- or low-angle rays, O- or X-mode polarization, E- or F-layer reflection, single- or multi-hop propagation). The impulse response of path *i* decomposes as

(2) $$h_{i}\left(t,\tau \right)=\sqrt{P_{i}\left(\tau \right)}\;e^{j\Phi_{i}\left(t,\tau \right)}\;M_{i}\left(t,\tau \right),$$

where $P_{i}\left(\tau \right)$ is the power delay profile (PDP), $e^{j\Phi_{i}\left(t,\tau \right)}$ is the deterministic phase term capturing Doppler-induced evolution, and $M_{i}\left(t,\tau \right)$ is a random modulation term introducing small-scale gain fluctuations. The complete channel impulse response for $N_{p}$ paths is

(3) $$h\left(t,\tau \right)=\sum_{i=1}^{N_{p}}\sqrt{P_{i}\left(\tau \right)}\;e^{j\Phi_{i}\left(t,\tau \right)}\;M_{i}\left(t,\tau \right).$$

In the improved model, each component is linked to the time-varying parameters from the perturbation module, enabling stochastic multipath channel modeling.

#### 2.3.1. Power Delay Profile

The PDP of each path is modeled by a Gamma distribution:

(4) $$P_{i}\left(\tau \right)=\frac{{\left(\tau -\tau_{c,i}\right)}^{\alpha_{i}-1}}{\Gamma \left(\alpha_{i}\right)\;\beta_{i}^{\alpha_{i}}}\exp\;\left(-\frac{\tau -\tau_{c,i}}{\beta_{i}}\right),\;\tau_{\min,i}\leq \tau \leq \tau_{\max,i},$$

where $\tau_{c,i}$ is a path-dependent delay shift, $\alpha_{i}>0$ is the shape parameter, $\beta_{i}>0$ is the scale parameter, Γ(·) is the Gamma function, and $\tau_{\min,i}$, $\tau_{\max,i}$ bound the support at the receiver sensitivity threshold [17]. When $\alpha_{i}=1$ the distribution reduces to an exponential, with energy concentrated near the earliest arrival; for $\alpha_{i}>1$, a peak forms around $\tau_{c,i}$, modeling a dominant delay with power spread on both sides [17]. Gamma PDPs have been used to describe wideband HF multipath delay profiles [17]; we adopt this form to represent a range of delay-spread behaviors without claiming realization-level fidelity to any specific measured channel. The parameters ($\alpha_{i}$, $\beta_{i}$, $\tau_{c,i}$) can be set from measured data or IRI-based calculations, linking the simulated delay characteristics to the underlying ionospheric state.

#### 2.3.2. Deterministic Phase Function

Ionospheric motion and disturbances introduce Doppler shifts, causing the channel phase to vary over time. For path *i* the instantaneous phase is approximated as

(5) $$\Phi_{i}\left(t,\tau \right)\approx 2\pi f_{D,i}\left(\tau \right)\;t+\Phi_{i,0}\left(\tau \right),$$

where $\Phi_{i,0}\left(\tau \right)$ is the initial phase and $f_{D,i}\left(\tau \right)$ is the associated Doppler shift. Different delay components may experience different Doppler shifts due to ionospheric drift at varying elevation angles. For simplicity, we assume all delay components of a given path share a common average shift $f_{D,i}$ with small random fluctuations to emulate Doppler spread. In a statistical sense, each path’s complex envelope $M_{i}\left(t,\tau \right)\;e^{j\Phi_{i}\left(t,\tau \right)}$ is modeled within a wide-sense stationary uncorrelated scattering (WSSUS) framework, under which distinct delay components are uncorrelated in the second-order sense [19]. If initial phases are independently and uniformly randomized, $E[e^{j\Phi_{i}\left(t,\tau \right)}]=0$ may hold; however, this is an additional modeling assumption rather than a direct consequence of WSSUS. The Doppler phase term allows $f_{D,i}$ to be adjusted to ionospheric conditions (e.g., irregularity drift rates), simulating scenarios from a quiescent ionosphere ($f_{D,i}\approx 0$) to a disturbed one with shifts of several hertz.

#### 2.3.3. Fast-Fading Random Modulation

Fast fading in HF channels refers to rapid amplitude fluctuations caused by multipath interference from small-scale ionospheric irregularities. The random modulation $M_{i}\left(t,\tau \right)$ is a stochastic process representing the time-varying gain of path *i* at delay τ. Its amplitude is commonly assumed to follow a Rayleigh distribution (or Rician if a dominant component is present) [24]. In all experiments we generate $M_{i}\left(t,\tau \right)$ via a filtered-noise method: a complex Gaussian white-noise sequence is passed through a filter whose frequency response is the square root of the target Doppler power spectrum, and the magnitude of the filtered process serves as the fading envelope. Because fading at closely spaced delays is approximately uncorrelated in practice, independent $M_{i}\left(t,\tau \right)$ processes are generated for discrete delay bins within each path. The autocorrelation of $M_{i}\left(t,\tau \right)$ can be configured based on measured Doppler spread and coherence time, reproducing fast-fading characteristics under different disturbance levels.

#### 2.3.4. Composite Channel Response

By integrating the three components above, the improved ITS model generates h(t,τ) at each time instant based on the current parameter set from the perturbation module. Path delays are no longer fixed: a path traversing the F2 layer, for example, experiences shorter delay at night due to ionospheric contraction and longer delay during daytime. This behavior is realized by dynamically adjusting the delay-offset parameters in $P_{i}\left(\tau \right)$ and the phase functions $\Phi_{i}$ through the perturbation module, while path gains evolve according to the dynamics of $M_{i}\left(t,\tau \right)$.

The resulting time-varying impulse response incorporates stochastic multipath fluctuations and captures key qualitative characteristics of ionospheric HF channels more faithfully than a static-parameter ITS model. In TDOA applications, random variation in path delays induces jitter in delay estimation; the proposed model thus enables investigation of algorithm robustness under realistic noise and ionospheric disturbance conditions.

To assess statistical plausibility, Table 1 compares key channel statistics from the proposed model against published HF sounding measurements over similar mid-latitude paths [16,17,18]. The simulated RMS delay spread, Doppler spread, and coherence bandwidth fall within the documented ranges, supporting the model’s use as a statistically representative testbed. This comparison is at the statistical level; the model is not intended to reproduce a specific measured instance, and residual mismatch is addressed by evaluating the estimator on both synthetic and real field data.

## 3. TDOA Estimation Method

The proposed TDOA estimation algorithm comprises three parallel branches integrating raw-waveform processing, correlation-feature extraction, and a CNN-based regression head. A raw-waveform branch applies a compact 1-D convolutional front end to learn timing cues directly from the signals. A multi-weight GCC branch computes PHAT, SCOT, and ML correlation features to capture robust inter-sensor delays. A lightweight channel-context branch aggregates descriptors such as SNR proxies, spectral flatness, and kurtosis to suppress spurious correlation peaks. The embeddings from the three branches are concatenated and fed to a fusion multilayer perceptron (MLP) (288→128→1) that outputs the estimated TDOA $\hat{\tau }$.

### 3.1. GCC-Based TDOA Modeling and Multi-Weight Feature Construction

Consider two synchronized receivers capturing a common source signal s[n] in the presence of channel effects and noise. Over a window of *N* samples, the received signals are

(6) $$x_{1}\left[n\right]=h_{1}\left[n\right]*s\left[n\right]+w_{1}\left[n\right],$$

(7) $$x_{2}\left[n\right]=h_{2}\left[n\right]*s\left[n\right]+w_{2}\left[n\right],$$

where $h_{1}\left[n\right]$ and $h_{2}\left[n\right]$ are the source-to-receiver impulse responses, $w_{1}\left[n\right]$ and $w_{2}\left[n\right]$ are modeled as zero-mean additive Gaussian white noise processes that are uncorrelated with the desired source signal, and ∗ denotes convolution. This AWGN assumption is adopted as a standard controlled baseline for TDOA modeling, simulation, and receiver-performance evaluation, and it has also been widely used in related HF geolocation simulation studies [11,13]. In practical HF skywave environments, however, the received disturbance may also contain external interference, impulsive components, and other colored or non-Gaussian contributions that are not explicitly represented in (6) and (7). To improve robustness against such model mismatch, the proposed estimator combines raw-waveform features, multi-weight GCC representations, and CSI-based conditioning, and its performance is further validated on field-recorded data without fine-tuning. The goal is to estimate the time delay τ between the two arrivals. In a direct-path scenario $h_{1}$ and $h_{2}$ reduce to delayed delta functions and τ equals the delay difference; in multipath scenarios, τ corresponds to the difference between the earliest significant arrivals.

The GCC method estimates τ by measuring the similarity between $x_{1}$ and time-shifted versions of $x_{2}$. The time-domain cross-correlation is

(8) $$R_{x_{1}x_{2}}\left(\tau \right)=\sum_{n=0}^{N-1}x_{1}\left[n\right]\;x_{2}^{*}\left[n+\tau \right],$$

where ${\left(\cdot \right)}^{*}$ denotes complex conjugation. In practice, GCC is computed via the cross-power spectrum. Let $X_{1}\left[k\right]=F\left\{x_{1}\left[n\right]\right\}$ and $X_{2}\left[k\right]=F\left\{x_{2}\left[n\right]\right\}$, where F{·} denotes the discrete Fourier transform. The cross-power spectral density is $G_{12}\left[k\right]=X_{1}\left[k\right]X_{2}^{*}\left[k\right]$. Applying a frequency-domain weighting Φ(k) and transforming back using the inverse discrete Fourier transform $F^{-1}\left\{\cdot \right\}$ yields the weighted GCC:

(9) $$R_{\Phi }\left(\tau \right)=F^{-1}\;\left\{\Phi \left(k\right)\;X_{1}\left[k\right]\;X_{2}^{*}\left[k\right]\right\}.$$

Different choices of Φ(k) produce GCC variants with distinct properties. The classic PHAT weighting $\Phi_{\mathrm{PHAT}}\left(k\right)=1/\left|G_{12}\left[k\right]\right|$ flattens the spectrum and sharpens the correlation peak, which is effective in reverberant and noisy conditions. SCOT and ML weighting target noise reduction and reverberation suppression, respectively.

The lag aperture $\left[-\tau_{\max},\tau_{\max}\right]$ is chosen to cover the plausible TDOA range. For the six-receiver configuration spanning approximately 2500 km in baseline, with ionospheric excess paths of up to 1000 km, we adopt $\tau_{\max}=0.02$ s (equivalent path difference 6000 km). At sampling rate $F_{s}=10,240$ Hz this yields D=2×(0.02×10,240)+1=409 discrete lag bins. The classical GCC delay estimate is

(10) $${\hat{\tau }}_{\mathrm{GCC}}=\underset{\tau }{\arg\max}\;R_{\Phi }\left(\tau \right).$$

In the proposed approach, multiple weightings $\left\{\Phi_{1},\Phi_{2},\ldots ,\Phi_{b}\right\}$ are applied to form a feature matrix $R\in R^{b\times D}$, where each row is the GCC waveform under a specific weighting. These weightings are standard; our contribution is to stack them as complementary correlation views whose individual reliability varies with noise and multipath conditions. In the absence of noise or multipath conditions, all $R_{\Phi_{i}}$ peak at the true delay; under challenging conditions the peak patterns diverge, but the collection helps a data-driven model identify the correct lag more robustly. All GCC maps are computed from full-sample signals, and the estimator operates on the multi-weight stack R.

### 3.2. CNN Architecture and Channel Enhancement Design

**(1) Network Overview.**

**Figure 2:**
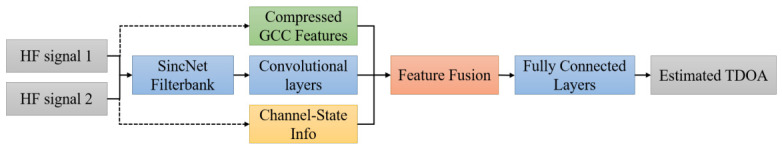
Block diagram of the proposed TDOA estimation CNN.

Figure 2 outlines the proposed architecture. The model adopts a three-branch design combining a physics-informed front end with correlation cues and context features. The raw-waveform branch uses a SincNet front end (64 filters, k=101, s=2, valid padding) followed by two 1-D convolutional blocks (k=7, s=2; then k=5, s=2; same padding) and a global average pooling (GAP) head yielding a 128-D embedding. The multi-weight GCC branch stacks PHAT, SCOT, and ML maps over D=409 lags and embeds them with a shallow CNN encoder (k=9, s=2; k=7, s=2; GAP) to produce another 128-D vector. The channel-state branch maps normalized CSI descriptors through a small MLP to a 32-D conditioning feature. The three outputs are concatenated into a fused vector of $128_{\mathrm{Sinc}}+128_{\mathrm{GCC}}+\left[32_{\mathrm{CSI}}\right]$, giving 288 dimensions with CSI and 256 without. A skip connection routes the SincNet output (after downsampling) toward the fusion pathway to preserve sub-sample timing cues; it is merged within the waveform branch before GAP, leaving the fusion dimensionality unchanged.

After fusion, a compact two-layer fully connected head (288→128→1, or 256→128→1 without CSI) with batch normalization (BN), ReLU, and dropout maps the fused vector to a continuous TDOA estimate. Each forward pass produces a single scalar delay for one receiver pair. For *M* receivers, the M−1 reference-based TDOAs required for localization are obtained by repeated pairwise inference. All components—SincNet cutoff frequencies, convolutional kernels, and fusion/head layers—are trained end to end with supervised regression objectives.

**(2) SincNet Front-End and Waveform Branch.**

Each receiver’s signal is represented as a complex-baseband stream $x_{m}\left[n\right]=I_{m}\left[n\right]+jQ_{m}\left[n\right]$ reconstructed from recorded I/Q samples. The two streams are processed by a SincNet-style front end [25] in which each filter is parameterized as a band-pass sinc kernel with only the low and high cutoff frequencies trainable. This reduces free parameters, enforces a physically interpretable filter structure, and improves training stability. We employ 64 filters (k=101, s=2, valid padding), each windowed by a Hamming function and initialized with logarithmically spaced cutoff frequencies to provide broad spectral coverage.

The SincNet layer is followed by two lightweight 1-D convolutional blocks. The first applies 64 filters (k=7, s=2, same padding) with BN, ReLU, and a pooling step; the second uses 128 filters (k=5, s=2, same padding) followed by GAP to produce a fixed 128-D embedding. Strided convolutions progressively reduce temporal resolution while preserving delay-discriminative structure. A skip connection routes the SincNet output (after downsampling) toward the fusion pathway before GAP, allowing the model to retain high-resolution timing cues in parallel with deeper abstractions.

**(3) GCC-Based Feature Branch.**

In parallel, a branch encodes cross-correlation information between the two channels. GCC functions are computed over the lag window $\left[-\tau_{\max},\tau_{\max}\right]$ with *D* samples using PHAT, SCOT, and ML weightings, forming a 3×D feature map. A shallow CNN encoder—two 1-D convolutional blocks (k=9 then k=7, stride 2, same padding, BN, ReLU) followed by GAP—compresses this map to a 128-D vector while suppressing spurious sidelobes. Under ideal conditions the GCC peak aligns with the true delay; in realistic HF channels the peak distribution across weightings still carries statistical evidence of the correct lag. Embedding these patterns in a learnable encoder allows the CNN to adapt its effective weighting strategy, improving resilience to multipath and low SNR.

**(4) Channel-State Information (CSI) Branch.**

An optional CSI branch provides auxiliary conditioning. It ingests two categories of descriptors: (i) signal-derived quantities—SNR proxy, carrier-frequency offset (CFO), effective bandwidth, spectral flatness, kurtosis, and sub-band signal-to-interference ratio (SIR)—estimated from the same receiver pair; and (ii) ionospheric state parameters ($f_{o}F_{2}$, $h_{mF2}$) obtained from the IRI-2020 model [26], which provides background ionospheric predictions from geographic coordinates, UTC time, solar flux F10.7, and the planetary Kp index. In an operational passive monitoring system, these IRI-derived parameters are intended as coarse environmental context rather than precise real-time local ionospheric measurements. This design is a practical compromise, because direct real-time acquisition of accurate local ionospheric state would require dedicated sounding infrastructure or equivalent external support, which would substantially increase system cost and deployment complexity. Under non-extreme space-weather conditions, the IRI-2020 empirical model generally provides stable large-scale ionospheric trends consistent with climatological behavior. Therefore, the resulting $f_{o}F_{2}$ and $h_{mF2}$ are used here as auxiliary conditioning features rather than hard physical constraints. When IRI predictions are unavailable or considered unreliable, missing entries are masked and zero-filled after z-score normalization, allowing the network to fall back to waveform and GCC evidence. The normalized CSI vector ($d_{CSI}=8$) is mapped to a 32-D embedding by a lightweight MLP (ReLU–dropout–ReLU). By conditioning on CSI the regressor can adjust its reliance on waveform versus correlation evidence according to the prevailing channel state.

**(5) Feature Fusion and Regression Head.**

The 128-D waveform embedding, 128-D GCC embedding, and 32-D CSI embedding (when used) are concatenated into a fusion vector of 288 (or 256 without CSI) dimensions. A two-layer fully connected head with BN, ReLU, and dropout (p=0.3) maps this vector to a single scalar delay estimate (288→128→1). All fusion and head layers are trained end to end jointly with the feature extractors.

**(6) Loss Function and Training Strategy.**

Given a training set ${\left\{\left(x_{i},\tau_{i}\right)\right\}}_{i=1}^{N}$, where $x_{i}$ denotes one receiver pair (waveforms, GCC features, and optional CSI) and $\tau_{i}$ is the ground-truth delay, the network predicts ${\hat{\tau }}_{i}=f_{\theta }\left(x_{i}\right)$. The training objective is a robust composite loss:

(11) $$L\left(\theta \right)=\alpha \cdot \mathrm{MSE}\left(\hat{\tau },\tau \right)+\left(1-\alpha \right)\cdot {\mathrm{Huber}}_{\delta }\left(\hat{\tau },\tau \right),$$

with α=0.5. Targets are in sample indices. The Huber threshold δ is set between 3 and 5 samples (≈0.29–0.49 ms at 10,240 Hz). An extended configuration additionally outputs a predicted variance $\sigma^{2}$ for heteroscedastic Gaussian NLL training; this is treated as an ablation and is not used for the main results.

Training uses the AdamW optimizer (learning rate $10^{-3}$, weight decay $10^{-4}$) with cosine annealing and a 5-epoch warmup. Mini-batch size is 32–64; gradient clipping at 1.0 stabilizes optimization. The model trains for up to 300 epochs with early stopping on validation loss (convergence is typically observed around epoch 150).

**(7) Model Complexity.**

In the baseline configuration of Table 2, the network comprises approximately 1.2 M trainable parameters, the majority residing in the waveform front end and temporal convolutions. On an NVIDIA RTX-series GPU ( PyTorch 2.7.0, FP32), a forward pass with batch size 32 takes under 20 ms for *T* = 15,360 samples, yielding an inference throughput exceeding 1.5 k windows per second. For embedded ARM processors, a pruned variant using depthwise separable convolutions reduces the count below 0.5 M and achieves under 50 ms per window.

For *M* receivers the deep-estimation cost scales linearly (M−1 forward passes under shared weights). The localization stage (Section 4) solves the SDP (29) once per update with a 4×4 lifted variable; its wall-clock time depends on the solver and the number of constraints rather than on the waveform sampling rate.

## 4. HF Skywave Geolocation via TDOA

Building on the TDOA estimates from Section 3, we develop a localization framework for HF skywave sources. Unlike conventional methods that rely on a fixed virtual reflection height, the proposed approach models the ionospheric excess path through receiver-specific nonnegative squared-range correction terms. This enables source localization without prior ionospheric information, addressing the challenge of time-varying and uncertain ionospheric conditions. By reformulating skywave propagation as a bias-augmented time-of-arrival (TOA) model, the estimation problem becomes amenable to convex optimization: we first formulate a bias-regularized constrained least-squares problem and then transform it into a semidefinite program (SDP) via convex relaxation.

### 4.1. Geometry and TDOA Measurement Model

We establish the geometric measurement model in a three-dimensional Earth-Centered, Earth-Fixed (ECEF) coordinate system under the WGS-84 ellipsoid. Consider an HF transmitter at unknown position $p\in R^{3}$ and *M* receivers at known positions $b_{i}\in R^{3}$ (i=1,…,M), all in the WGS-84 ECEF frame with a common time base. Inter-receiver clock offsets within one TDOA window are assumed negligible; absolute synchronization to the transmitter is unnecessary because the unknown transmit time $t_{0}$ is eliminated by differencing.

We adopt a single-hop skywave model (one dominant ionospheric reflection) as the baseline propagation geometry, targeting scenarios in which a single reflection mode dominates or in which the earliest resolvable arrival is selected for TDOA estimation. When comparable multi-hop components exist, the excess-path term may no longer admit a strict single-reflection interpretation, and localization accuracy may degrade; explicit multi-hop modeling is beyond the scope of this paper.

Let $r_{i}$ denote the total propagation distance from p to receiver $b_{i}$. The geometric line-of-sight (LOS) distance is

(12) $$d_{i}={∥p-b_{i}∥}_{2}.$$

where ${∥\cdot ∥}_{2}$ denotes the Euclidean norm. Due to ionospheric reflection, the actual path includes an unknown excess distance $e_{i}\geq 0$:

(13) $$r_{i}=d_{i}+e_{i}+n_{i}=c\;\Delta t_{i0},\;i=1,\ldots ,M,$$

where *c* is the speed of light, $\Delta t_{i0}=t_{i}-t_{0}$ with $t_{i}$ the arrival time at receiver *i*, and $n_{i}$ is zero-mean Gaussian measurement noise in the distance domain. The excess path is

(14) $$e_{i}=\;∥p-s_{i}{∥}_{2}+\;∥s_{i}-b_{i}{∥}_{2}-\;{∥p-b_{i}∥}_{2},$$

where $s_{i}$ is the (unknown) ionospheric reflection point. Under multi-hop propagation, $e_{i}$ retains its interpretation as a nonnegative aggregate excess path, though the single-point interpretation of $s_{i}$ is no longer exact. In this paper, $e_{i}$ is treated as a receiver-specific constant within each localization update and is estimated jointly with p; within-window variability is absorbed into the noise term.

Receiver $b_{1}$ is designated as the reference with $t_{1}=0$. The measured TDOA relative to the reference is $\Delta t_{i1}=t_{i}-t_{1}$, so $t_{i}=\Delta t_{i1}$ for i≥2 and only i≥2 contribute to the data-fidelity objective. We assume that all TDOA estimates are extracted from a common signal segment corresponding to the same transmit epoch $t_{0}$. Although the correction terms $\left\{u_{i}\right\}$ are receiver-specific, they do not create independent unconstrained range equations because all non-reference receivers share the same unknowns p and $t_{0}$, and the feasible set is further restricted by the constraints below.

A basic causality constraint requires

(15) $$t_{i}\geq t_{0},\;i=1,\ldots ,M.$$

Since $e_{i}\geq 0$, the measured path must be no shorter than the LOS distance:

(16) $$\left(t_{i}-t_{0}\right)\;c\geq d_{i},\;i=1,\ldots ,M.$$

By the triangle inequality, for any receiver pair (i,j):

(17) $$\left(t_{i}-t_{0}\right)\;c+\left(t_{j}-t_{0}\right)\;c\geq {∥b_{i}-b_{j}∥}_{2},\;\forall \;i\neq j.$$

These constraints eliminate unphysical candidate positions and remain conservative under multi-hop propagation, though they become looser as $e_{i}$ increases. In practice, strict inequalities are approximated by adding a small margin ϵ>0.

### 4.2. Bias-Regularized Constrained Least-Squares Formulation

The goal is to jointly estimate p, $t_{0}$, and nonnegative squared-range correction terms so that the modeled TDOAs match the observations. From (13), the distance-domain residual for receiver *i* is

(18) $$\rho_{i}≜c\;\left(t_{i}-t_{0}\right)-d_{i}-e_{i}.$$

In the noise-free case $\rho_{i}=0$; under noise $\rho_{i}=n_{i}$. Applying the difference-of-squares identity:

(19) $$c^{2}{\left(t_{i}-t_{0}\right)}^{2}-{\left(d_{i}+e_{i}\right)}^{2}=2\;n_{i}\left(d_{i}+e_{i}\right)+n_{i}^{2}.$$

We introduce the auxiliary variable

(20) $$u_{i}≜e_{i}^{2}+2\;d_{i}\;e_{i},$$

so that ${\left(d_{i}+e_{i}\right)}^{2}=d_{i}^{2}+u_{i}$. Substituting into (19) with $d_{i}={∥p-b_{i}∥}_{2}$ yields

(21) $$c^{2}{\left(t_{i}-t_{0}\right)}^{2}-d_{i}^{2}-u_{i}=2\;n_{i}\left(d_{i}+e_{i}\right)+n_{i}^{2}.$$

The substitution (20) is an exact algebraic reparameterization and does not assume small noise. The approximation enters when the left-hand side, $g_{i}≜c^{2}{\left(t_{i}-t_{0}\right)}^{2}-d_{i}^{2}-u_{i}$, is treated as a residual to be driven toward zero. Under noise, (21) shows $g_{i}=2n_{i}\left(d_{i}+e_{i}\right)+n_{i}^{2}\neq 0$ in general; accordingly, we minimize the aggregate mismatch rather than enforcing $g_{i}=0$.

This leads to the constrained least-squares formulation:

(22) $$\begin{array}{cc} \min_{p,\;t_{0},\;\left\{u_{i}\right\}} & F=\sum_{i=2}^{M}w_{i}{\left(c^{2}{\left(t_{i}-t_{0}\right)}^{2}-{∥p-b_{i}∥}_{2}^{2}-u_{i}\right)}^{2}\\ s.t. & t_{i}\geq t_{0},\;i=2,\ldots ,M,\\ \; & c\;\left(t_{i}-t_{0}\right)\geq {∥p-b_{i}∥}_{2},\;i=2,\ldots ,M,\\ \; & c\;\left(t_{i}-t_{0}\right)+c\;\left(t_{j}-t_{0}\right)\geq {∥b_{i}-b_{j}∥}_{2},\;\forall \;i\neq j,\\ \; & u_{i}\geq 0,\;i=2,\ldots ,M, \end{array}$$

where $w_{i}>0$ are reliability weights. In all reported experiments we use uniform weights $w_{i}=1$ for all methods. The objective sums only over i=2,…,M because receiver 1 serves as the time origin.

Minimizing the time-domain residual $\rho_{i}=c\left(t_{i}-t_{0}\right)-d_{i}-e_{i}$ directly involves the nonconvex norm $d_{i}$ and an explicit coupling with the non-line-of-sight (NLOS) bias $e_{i}$, producing bilinear terms that are difficult to convexify. By moving to the squared-range domain and introducing $u_{i}$, the NLOS contribution is absorbed into a single nonnegative variable and the residual becomes polynomial in the decision variables, enabling the SDP relaxation derived next.

### 4.3. SDP-Based Convex Relaxation

Problem (22) is nonconvex due to the quadratic dependence on $t_{0}$ and the coupling among p, $d_{i}$, and $u_{i}$. We relax it into a convex SDP through three steps.

**Step 1: Lifting the squared range.** We introduce $h_{i}$ as a surrogate for $d_{i}^{2}={∥p-b_{i}∥}_{2}^{2}$ and define the lifted PSD matrix

(23) $$M=\left[\begin{array}{cc} I_{3} & p\\ p^{T} & o \end{array}\right]⪰0,$$

where *o* is a scalar lifting variable. Defining $a_{i}={\left[b_{i}^{T},\;-1\right]}^{T}$, we obtain

(24) $$h_{i}=a_{i}^{T}M\;a_{i}=o-2\;b_{i}^{T}p+{∥b_{i}∥}_{2}^{2}.$$

Since M⪰0 implies $o\geq {∥p∥}_{2}^{2}$, we have

(25) $$h_{i}=∥p-b_{i}{∥}_{2}^{2}+\left(o-{∥p∥}_{2}^{2}\right)\;\geq \;{∥p-b_{i}∥}_{2}^{2}.$$

Equality holds when $o={∥p∥}_{2}^{2}$ (rank-one solution); otherwise $h_{i}$ provides a convex upper bound on the true squared range.

**Step 2: Retaining $u_{i}$ as a nonnegative surrogate.** The quantity $u_{i}=e_{i}^{2}+2d_{i}e_{i}\geq 0$ from (20) is retained as a nonnegative effective squared-range correction coupled to $\left(p,t_{0}\right)$ through the shared residual and geometric constraints. It should be interpreted as an excess-path surrogate in the squared-range domain rather than as a uniquely recoverable physical parameter.

**Step 3: Convexifying the dependence on $t_{0}$.** We introduce *q* with

(26) $$\left(\begin{array}{cc} 1 & t_{0}\\ t_{0} & q \end{array}\right)⪰0\;⟹\;q\geq t_{0}^{2}.$$

Each residual then becomes affine in the decision variables:

(27) $$g_{i}≜c^{2}t_{i}^{2}+c^{2}q-2c^{2}t_{i}t_{0}-h_{i}-u_{i}.$$

We introduce nonnegative slack variables $s_{i}\geq 0$ satisfying

(28) $$-s_{i}\leq g_{i}\leq s_{i},\;i=2,\ldots ,M,$$

so that the data-fidelity term is rewritten in epigraph form as $\sum_{i}w_{i}s_{i}^{2}$ subject to $|g_{i}|\;\leq s_{i}$.

**Final SDP formulation.** All nonconvexities are now relaxed into LMIs, SOC constraints, and convex quadratic terms:

(29) $$\begin{array}{cc} \min_{\begin{array}{c} p,\;t_{0},\;q,\\ \left\{h_{i}\right\},\left\{u_{i}\right\},\left\{s_{i}\right\},o \end{array}} & \;\sum_{i=2}^{M}w_{i}s_{i}^{2}+\lambda \sum_{i=2}^{M}u_{i}^{2}\\ s.t. & t_{i}\geq t_{0},\;i=1,\ldots ,M,\\ \; & ∥p-b_{i}{∥}_{2}\;\leq c\;\left(t_{i}-t_{0}\right)-\epsilon ,\;i=1,\ldots ,M,\\ \; & c\;\left(t_{i}-t_{0}\right)+c\;\left(t_{j}-t_{0}\right)\geq {∥b_{i}-b_{j}∥}_{2}+\epsilon ,\;\forall \;i\neq j,\\ \; & h_{i}=o-2\;b_{i}^{T}p+{∥b_{i}∥}_{2}^{2},\;i=1,\ldots ,M,\\ \; & \left[\begin{array}{cc} I_{3} & p\\ p^{T} & o \end{array}\right]⪰0,\;\left(\begin{array}{cc} 1 & t_{0}\\ t_{0} & q \end{array}\right)⪰0,\\ \; & -s_{i}\leq g_{i}\leq s_{i},\;s_{i}\geq 0,\;u_{i}\geq 0,\;i=2,\ldots ,M. \end{array}$$

Here ϵ>0 is a small numerical margin (fixed at ϵ=1 m in all experiments) and λ>0 is the Tikhonov regularization weight on the excess-path surrogates (fixed at $\lambda =10^{-14}$). The first objective term $\sum w_{i}s_{i}^{2}$ is a convex surrogate of the weighted squared residuals; the second term $\lambda \sum u_{i}^{2}$ penalizes large surrogate corrections to stabilize the fit. In all reported experiments both $w_{i}$ and λ are kept identical across methods. The constraint $h_{i}=o-2b_{i}^{T}p+{∥b_{i}∥}_{2}^{2}$ together with M⪰0 ensures $h_{i}\geq {∥p-b_{i}∥}_{2}^{2}$, providing a consistent squared-range surrogate inside $g_{i}$, while the physical range bound is enforced directly by the SOC constraint.

The optimization problem in (29) is a convex conic program (SDP + SOC) solvable by interior-point methods. The source position p=(x,y,z) in the WGS-84 ECEF frame is read directly from the relaxed solution. Because the PSD lifting does not guarantee exact tightness under noise, we monitor the diagnostic gap

(30) $$\Delta_{\mathrm{tight}}≜o-{∥p∥}_{2}^{2},$$

which indicates how far the solution departs from the rank-one ideal. All reported geolocation results are taken directly from the relaxed solution without post-SDP local refinement or tightness-based filtering.

## 5. Simulation and Experimental Results

### 5.1. Training Data Preparation

The proposed TDOA network is trained on synthetically generated HF skywave receiver-pair samples. Each sample consists of two received signals from the same HF transmission, simulated as AM waveforms with center frequency $f_{c}=7.23$ MHz and bandwidth B=5 kHz. Each receiver stream is a complex-baseband sequence $x_{m}\left[n\right]=I_{m}\left[n\right]+jQ_{m}\left[n\right]$, so one network input corresponds to one synchronized receiver pair. The two-channel signals are generated from the channel model in Section 2. For computational efficiency, each receiver-link realization retains only the dominant earliest-arriving component and the strongest delayed component; the waveform generator should therefore be interpreted as a reduced-order instantiation of the full model. The resulting TDOA, determined by the sample geometry, serves as the supervised regression target.

Waveforms are sampled at $F_{s}=10,240$ Hz with duration 1.5 s. Additive white Gaussian noise (AWGN) is injected with SNR uniformly drawn from −10 to 20 dB. Lightweight augmentation—random temporal jitter, small carrier-frequency offsets, and mild band-limiting filters—is applied to improve robustness to modeling mismatch. All geographic coordinates are converted to ECEF (WGS-84) and all norms are computed in meters. Each waveform is normalized per-receiver in the complex domain to equalize scale while preserving relative temporal structure and phase.

Feature extraction follows the proposed architecture: one branch passes the waveform pair through the SincNet front end; the other computes GCC features over a lag window of ±0.02 s (D=409 lags at $F_{s}=10,240$ Hz), chosen to cover the maximum plausible TDOA under the six-receiver geometry (baseline ≈2500 km) with margin for ionospheric excess paths. Optional CSI descriptors are extracted from the same observation.

The full corpus contains $N_{\mathrm{total}}=120,000$ samples, split 80%/10%/10% into training (96,000), validation (12,000), and test (12,000) subsets. The test subset uses independent random seeds with new channel realizations and signal content, ensuring that no test sample is seen during training or validation.

### 5.2. Performance Metrics

Unless otherwise stated, delay errors are converted to equivalent range errors via $\Delta r\;\left(\mathrm{km}\right)≜\frac{c}{1000}\left|\hat{\tau }-\tau \right|$.

**(1) TDOA accuracy: MAE.** The mean absolute error in equivalent range units:

(31) $$\mathrm{MAE}=\frac{1}{N}\sum_{i=1}^{N}\frac{c}{1000}\left|{\hat{\tau }}_{i}-\tau_{i}\right|\;\left(\mathrm{km}\right).$$

**(2) Training/validation monitoring: RMSE.** The root-mean-square error is used only internally to monitor convergence:

(32) $${\mathrm{RMSE}}_{\mathrm{TDOA}}=\sqrt{\frac{1}{N}\sum_{i=1}^{N}{\left({\hat{\tau }}_{i}-\tau_{i}\right)}^{2}}\;\left(s\right).$$

**(3) Theoretical reference: CRLB.** Under the idealized single-path AWGN model with bandwidth B=5 kHz and $F_{s}=10,240$ Hz, the Cramér–Rao lower bound (CRLB) for unbiased delay estimation is

(33) $$\mathrm{var}\left(\hat{\tau }\right)\geq \frac{1}{8\pi^{2}\beta_{2}\;\mathrm{SNR}},$$

where $\beta_{2}$ is the second spectral moment of the baseband waveform. The equivalent-range bound is $\Delta r_{\mathrm{CRLB}}=\frac{c}{1000}\sqrt{\mathrm{var}(\hat{\tau })}$. This CRLB serves as an optimistic reference, since it does not account for multipath or excess-delay bias.

**(4) End-to-end localization: geolocation MAE.** To distinguish TDOA-level and position-level errors, we define the geolocation MAE as the mean Euclidean distance between estimated and true positions:

(34) $${\mathrm{MAE}}_{\mathrm{Geo}}=\frac{1}{N}\sum_{i=1}^{N}{∥{\hat{p}}_{i}-p_{i}∥}_{2}\;\left(\mathrm{km}\right).$$

Unless otherwise stated, the MAE–SNR curves in this section refer to ${\mathrm{MAE}}_{\mathrm{Geo}}$. The TDOA-level MSE shown in convergence plots reflects only the regression error and is not directly comparable.

### 5.3. Validation on Synthetic Data

**Figure 3:**
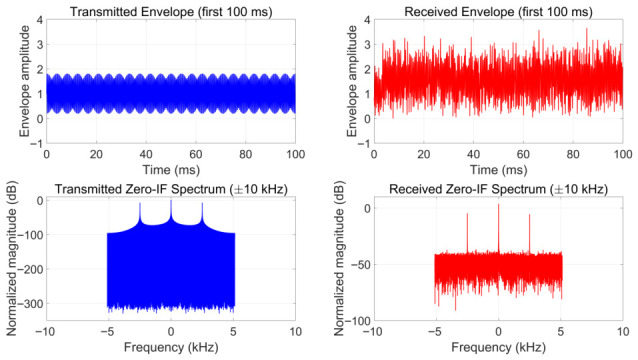
Representative synthetic HF signal: transmitted envelope and zero-IF spectrum (**left**) versus received counterparts after ionospheric propagation at SNR = 10 dB (**right**).

The trained network is evaluated on the held-out synthetic test set with new channel realizations. Performance is assessed over SNR levels from −10 to 20 dB. Figure 3 shows a representative transmitted signal and the corresponding received signal after ionospheric propagation at SNR = 10 dB. We compare the proposed estimator against GCC-PHAT, FS-GCC [27], and MATE [28], with the CRLB as an optimistic reference.

**Figure 4:**
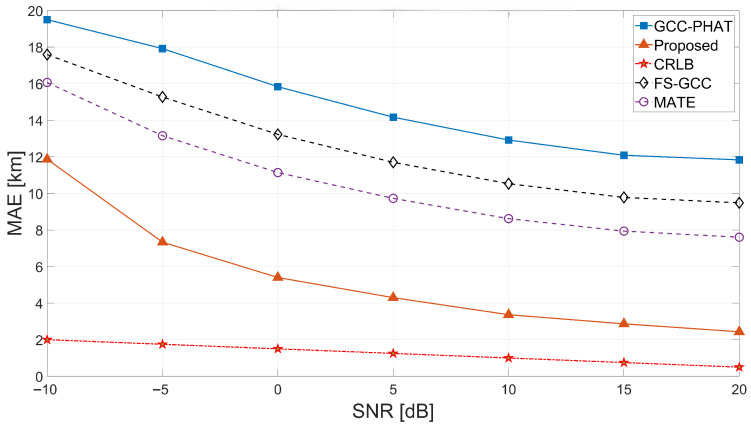
TDOA MAE in equivalent range (km) versus SNR for the proposed method and baselines.

**TDOA MAE vs. SNR.** Figure 4 plots the MAE in equivalent range versus SNR. The proposed method decreases monotonically from ≈12 km at −10 dB to ≈2.6 km at 20 dB, consistently outperforming GCC-PHAT (>12 km even at high SNR). FS-GCC decreases from ≈17.7 to ≈9.5 km, and MATE from ≈16.0 to ≈7.6 km. The proposed curve remains markedly closer to the CRLB, and the gap narrows with increasing SNR, consistent with the expected behavior under the assumed noise model. At 20 dB, the proposed MAE of 2.6 km is within a factor of approximately 2.5 of the CRLB-equivalent range (1 km), whereas GCC-PHAT remains more than an order of magnitude above the bound.

**Figure 5:**
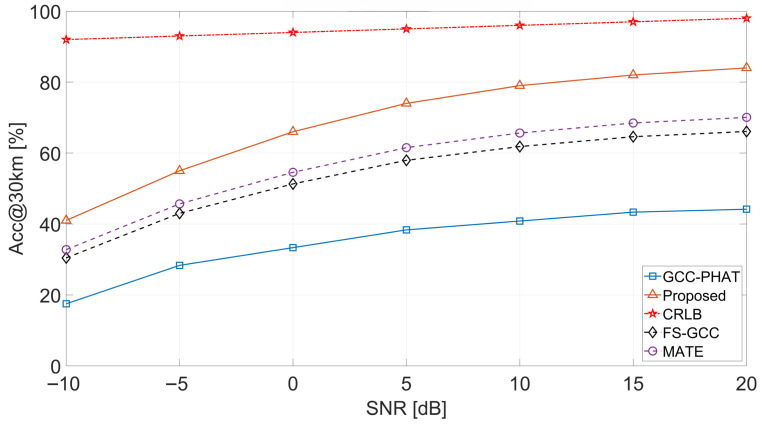
Acc@30 km versus SNR for the proposed method and baselines.

**Acc@30 km vs. SNR.** Figure 5 reports the probability that the TDOA equivalent-range error falls within 30 km:

(35) $$\mathrm{Acc}@30\;\mathrm{km}≜\mathrm{Pr}\;\left(\frac{c}{1000}\left|\hat{\tau }-\tau \right|\leq 30\;\mathrm{km}\right).$$

The proposed estimator rises from ≈41% at −10 dB to ≈84% at 20 dB, whereas GCC-PHAT reaches only ≈44%. MATE and FS-GCC attain ≈70% and ≈66% at 20 dB, respectively. The CRLB reference stays near 93–99%.

**Ablation study.** Table 3 quantifies the contribution of each branch on the synthetic test set. Each variant is retrained independently with grid search over learning rate $\in \{10^{-4},10^{-3},10^{-2}\}$ and dropout rate ∈{0.2,0.3,0.5}. All metrics are averaged over 12,000 samples spanning −10 to 20 dB.

Removing CSI increases TDOA MAE from 10.2 to 15.8 μs (+55%), confirming that channel-state descriptors enable the network to adaptively weight waveform and correlation evidence. The waveform-only variant achieves 18.4 μs (+80%), retaining delay-discriminative phase cues but lacking robustness under severe multipath. The GCC-only variant yields 24.6 μs (+141%). Although the multi-weight GCC branch already outperforms classical single-weight GCC-PHAT (Figure 4), it relies solely on correlation-domain evidence and cannot exploit the fine-grained phase structure present in the raw waveforms; the absence of both waveform cues and channel-state conditioning limits its ability to resolve ambiguous or shifted correlation peaks under severe multipath. Acc@30 km drops from 68.5% (full) to 44.2% (GCC-only), indicating that roughly one-third of challenging cases are resolved only through multi-modal fusion.

**Figure 6:**
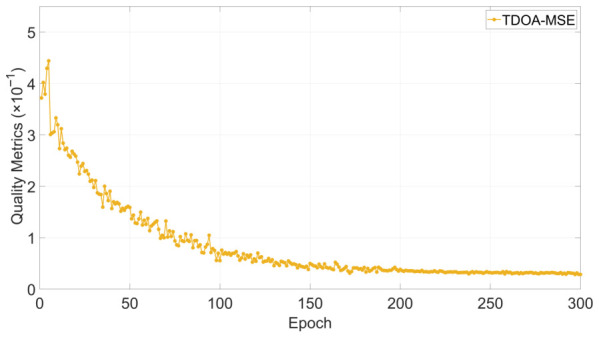
Training convergence: validation TDOA-MSE versus epoch.

**Training convergence.** Figure 6 shows the validation MSE curve versus epoch. The MSE drops sharply in the early epochs and gradually plateaus after approximately 150 epochs, indicating stable convergence under the adopted configuration.

**End-to-end geolocation on synthetic data.** At the TDOA level, the proposed estimator is compared with GCC-PHAT, FS-GCC, and MATE. At the geolocation level, the proposed framework is further compared with three representative localization baselines, namely ANN-TDOA [9], Improved-TDOA [14], and the method in [10] (denoted as TDOA-SDP). For each Monte Carlo trial the receiver coordinates, transmitter location, channel realization, and noise instance are identical across methods. All experiments use CVX with MOSEK, uniform weights $w_{i}=1$, ϵ=1 m, $\lambda =10^{-14}$, and tightness threshold $\tau_{\mathrm{tight}}=10^{6}$ m^2^. No post-SDP refinement is applied.

Six receivers are deployed at Beijing (116.26° E, 39.66° N), Chengdu (103.88° E, 30.66° N), Wuyishan (118.08° E, 27.46° N), Shanghai (121.58° E, 30.87° N), Kunming (102.95° E, 24.62° N), and Shenzhen (114.32° E, 22.64° N).

**Figure 7:**
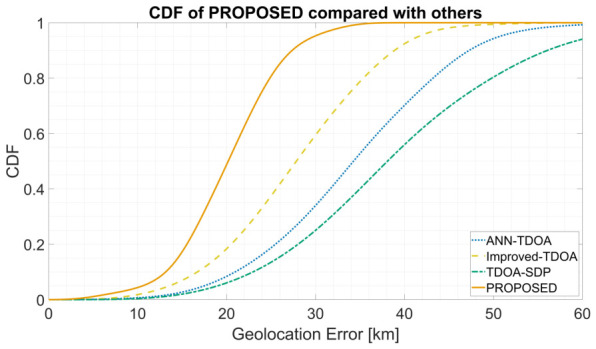
CDF of end-to-end geolocation error for the proposed method and three baselines on synthetic data.

**Figure 8:**
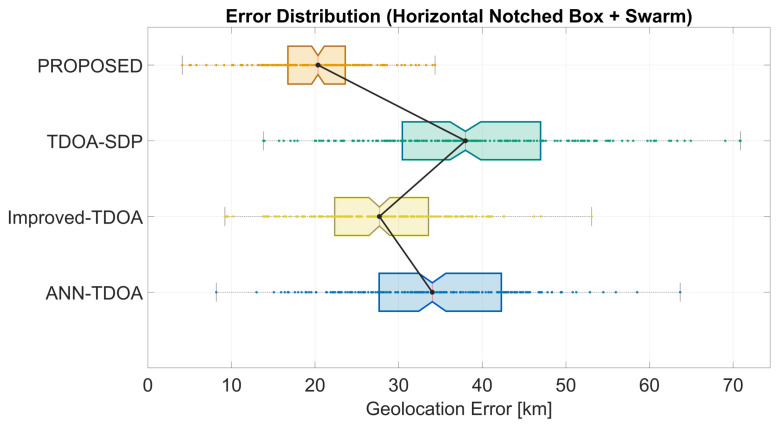
Notched box plots with swarm overlays of geolocation error on synthetic data. Notch centers indicate medians; box edges indicate the interquartile range.

Figure 7 shows the CDF of end-to-end geolocation error. The proposed method’s CDF rises earlier and more steeply, reaching high success probability within the 30 km regime, whereas the baselines require noticeably larger thresholds. Figure 8 presents horizontal notched box plots with swarm overlays: the proposed method exhibits the lowest median, the most compact interquartile range, shorter whiskers, and fewer large-error outliers. In particular, the proposed curve reaches approximately 80% cumulative probability at the 30 km threshold, whereas the best-performing baseline (TDOA-SDP) requires roughly 40 km to attain the same level. The box plot further reveals that the proposed distribution has a noticeably weaker long-tail behavior, with the swarm points concentrated below 25 km and fewer instances exceeding 40 km, suggesting improved robustness against occasional extreme errors in addition to better central accuracy.

**Figure 9:**
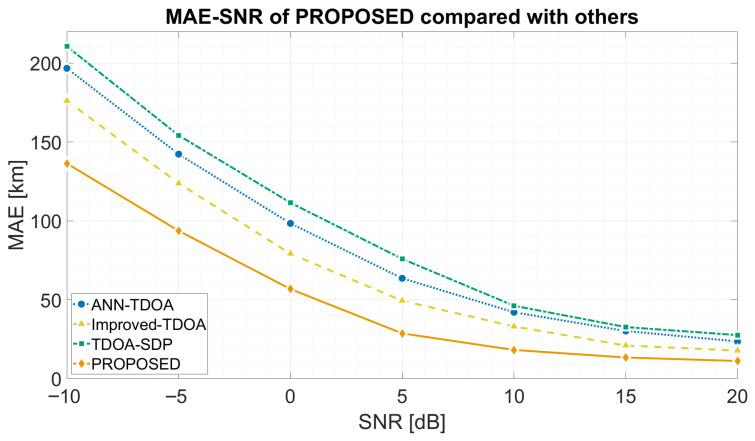
${\mathrm{MAE}}_{\mathrm{Geo}}$ (km) versus SNR for the proposed method and three baselines.

Figure 9 plots ${\mathrm{MAE}}_{\mathrm{Geo}}$ versus SNR. All methods decrease monotonically with SNR; the proposed method consistently attains the lowest error. At −10/0/20 dB the proposed method achieves 136.4/56.8/11.2 km, versus 176.1/79.2/17.8 km for Improved-TDOA, corresponding to a 20–40% reduction. ANN-TDOA yields 200.5/98.4/18.6 km at the same SNR points, and TDOA-SDP yields 185.2/82.1/15.3 km, confirming that the proposed method maintains a consistent advantage across all compared baselines and SNR levels.

**Figure 10:**
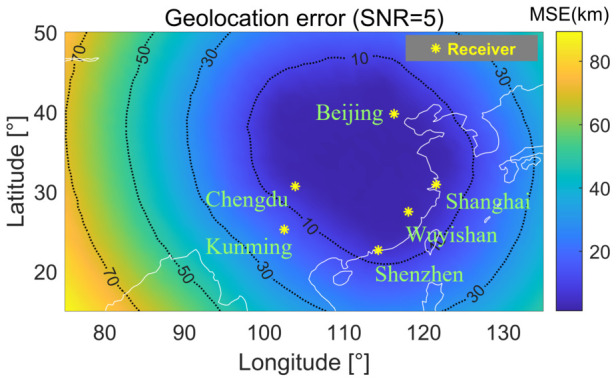
Geolocation error heatmap at 5 dB SNR (six-receiver configuration). Green stars: receivers; contours: iso-error levels.

Figure 10 maps the spatial variation of geolocation error at SNR = 5 dB over a uniform latitude–longitude grid. A pronounced low-error basin forms in the central region supported by the receiver geometry, with errors in the 10–30 km range and values as low as ≈8 km near the geometric centroid of the array. Error increases progressively toward the periphery, reaching ≈70–90 km at the western and northern boundaries where the source lies well outside the receiver enclosure. This degradation pattern is consistent with reduced geometric diversity and a larger geometric dilution of precision (GDOP) outside the receiver polygon.

### 5.4. Validation on Field Data

**Dataset description.** The estimator was validated on 100 signal segments from an AM broadcast source at Xi’an ($108.61^{{}^{\circ}}$ E, $34.37^{{}^{\circ}}$ N), received by six GPS-synchronized HF receivers in Beijing, Chengdu, Shanghai, Shenzhen, Wuyishan, and Kunming. Baseband I/Q data were recorded at $F_{s}=10,240$ Hz with $f_{c}=7.23$ MHz and B=5 kHz. Each receiver was equipped with a GPS-disciplined oscillator ensuring sub-microsecond synchronization. The 100 segments are non-overlapping 1.5 s windows (N=15,360 samples) from a single recording session (09:15–09:40 UTC, 24 June 2024) with average inter-segment spacing of ≈15 s. Although consecutive segments are not strictly independent (the mid-latitude HF coherence time is 60–90 s under moderate geomagnetic activity), the 25-min window spans multiple coherence intervals and provides meaningful statistical diversity.

**Reference TDOAs.** Benchmark TDOA references were constructed from header-derived arrival-time markers differenced across receivers. This reference is signal-derived rather than externally calibrated and is therefore used as a benchmark, not exact ground truth. A sanity check against GCC-PHAT peaks on high-SNR segments showed disagreement typically within ±5 km for 92% of checked cases. Accordingly, the benchmark itself carries a residual uncertainty on the order of a few kilometers, and the TDOA deviations reported in Table 4 and Table 5 should therefore be interpreted as benchmark-referenced errors rather than absolute ground-truth errors. This uncertainty may slightly perturb the benchmark-referenced TDOA-error values and may blur very small inter-method differences. However, it does not directly determine the end-to-end geolocation error statistics reported below, because those geolocation errors are computed against the known transmitter coordinates at Xi’an rather than against the benchmark TDOAs.

**Ionospheric conditions.** IRI-2020 retrievals at the Xi’an–receiver midpoints yield $f_{o}F_{2}\approx 8.2$ MHz and $h_{\mathrm{mF}2}\approx 285$ km (Kp ≈ 3), indicating moderately disturbed daytime conditions under which F-layer reflection is plausible, although weaker higher-order contributions may be present. Waveforms were band-limited to ±2.5 kHz to match the synthetic training data. The model was applied in zero-shot mode (no fine-tuning on field data). Beijing serves as the reference receiver; the five pairs (Chengdu–, Shenzhen–, Shanghai–, Wuyishan–, Kunming–Beijing) are evaluated per segment.

**Figure 11:**
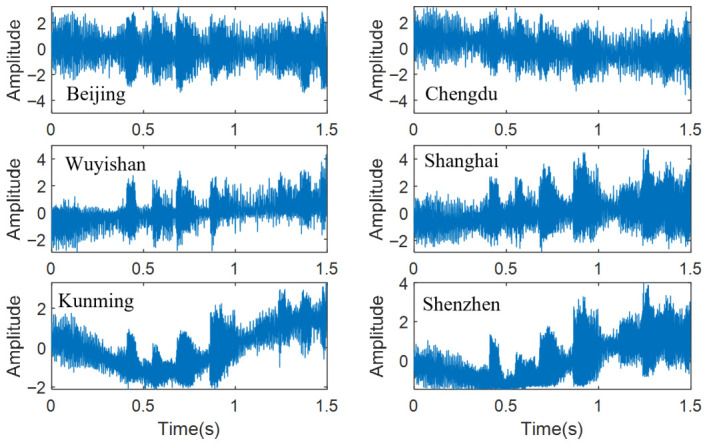
Representative field HF signal segment. Source: Xi’an (108.61° E, 34.37° N); six receivers; $F_{s}=10,240$ Hz; 09:15 UTC, 24 June 2024.

**Single-segment illustration.** Figure 11 shows a representative received waveform at the six receivers, illustrating the multipath-induced envelope distortion and differential arrival times across the array. Table 4 reports TDOA estimates and benchmark-referenced absolute deviations for all five receiver pairs on one segment. The proposed method achieves a mean deviation of 18.3 km, versus 35.5 km (GCC-PHAT), 34.4 km (FS-GCC), and 31.1 km (MATE). Improvements are consistent across all five receiver pairs, with the largest absolute reduction observed on the Wuyishan–Beijing link (from 39.3 km for GCC-PHAT to 15.8 km for the proposed method). Removing the CSI branch degrades the proposed method to 28.1 km, confirming the contribution of channel-state conditioning even on a single-segment basis.

**Figure 12:**
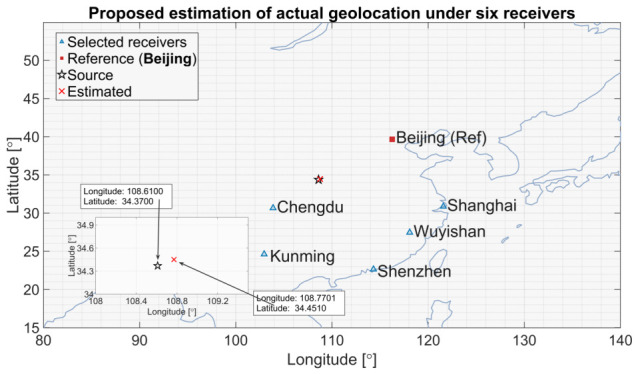
Single-segment geolocation. Black star: true source at Xi’an; red cross: proposed estimate (17.2 km error). Inset magnifies the source region. Green stars: receivers.

End-to-end geolocation on this segment yields 17.2 km for the proposed method, versus 42.8 km (GCC-PHAT), 51.3 km (FS-GCC), 48.6 km (MATE), 38.5 km (ANN-TDOA), 29.7 km (Improved-TDOA), and 24.1 km (TDOA-SDP). Removing CSI degrades the result to 27.5 km. This demonstrates that improved TDOA accuracy directly propagates to enhanced positioning performance, and that the multi-branch fusion architecture with channel-state conditioning achieves superior geolocation accuracy compared to both classical correlation methods and alternative deep learning approaches. Figure 12 shows the geographic layout and the estimated source location.

**One-hundred-segment TDOA performance.** Table 5 summarizes the benchmark-referenced TDOA deviations over all 100 segments. Averaged across the five receiver pairs, the proposed method achieves 20.3 km, a 51% reduction relative to GCC-PHAT (41.6 km). Notably, GCC-PHAT exhibits large inter-pair variability (25.5 km for Chengdu versus 48.2 km for Shenzhen), whereas the proposed method yields more uniform performance across all five links (17.4–22.2 km), suggesting that the learned fusion strategy is less sensitive to link-specific propagation geometry than classical correlation methods. Removing CSI increases the error to 31.7 km (+56%), supporting the practical value of channel-state conditioning. FS-GCC and MATE yield intermediate errors of 39.6 and 34.8 km, respectively. The single-segment example (Table 4) yields slightly lower error (18.3 km) than the 100-segment average (20.3 km), consistent with normal statistical variation across the dataset. The field-data error exceeds the synthetic performance at comparable SNR, because the simulation adopts a controlled AWGN-based disturbance model for tractable benchmarking, whereas practical HF environments may additionally contain external interference, impulsive components, and other non-Gaussian or colored effects. Thus, the synthetic results mainly verify feasibility and support fair comparison under the same controlled conditions, while the field experiments demonstrate the practical effectiveness of the proposed method under real-world conditions.

**Figure 13:**
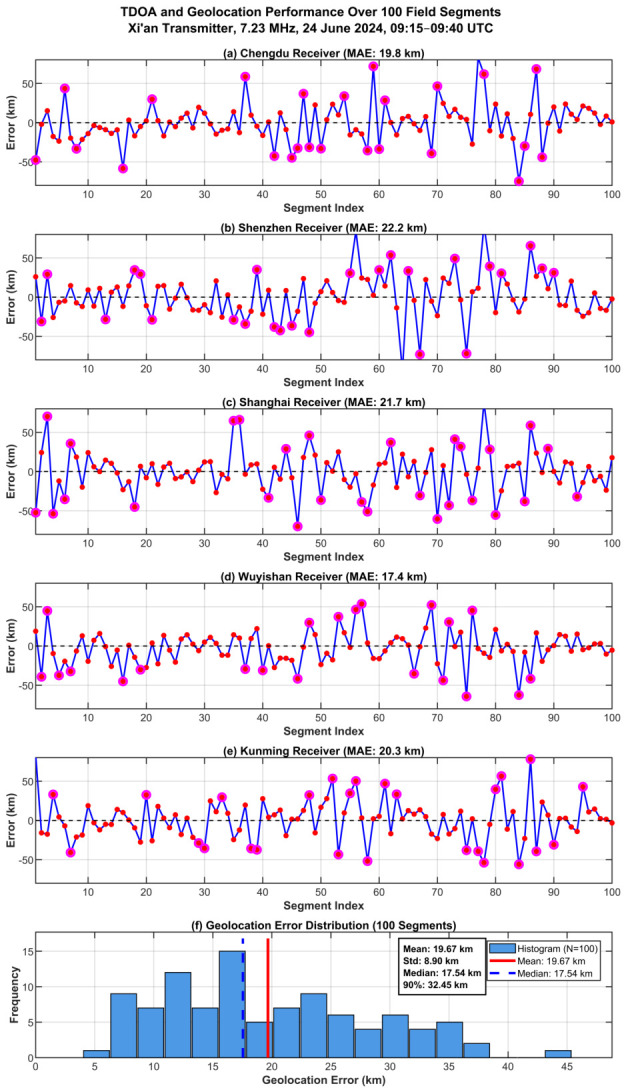
Performance over 100 field segments. (**a**–**e**) TDOA error time series for the five receiver pairs relative to Beijing; magenta circles: errors exceeding 28 km. (**f**) Geolocation error distribution.

**Temporal error analysis.** Figure 13a–e presents the TDOA prediction errors across the 100 segments for all five receiver pairs. The time series exhibit zero-mean fluctuations consistent with the per-pair MAE values in Table 5. Large-error events (magenta circles) occur in temporally clustered patterns (e.g., segments 14–17, 48–52, 77–80, 94–97), consistent with the expectation that ionospheric degradation persists over multiple observation windows. These clusters are broadly aligned across receiver pairs, suggesting a common environmental contribution. No sustained drift is observed over the 25-min window.

**Geolocation error distribution.** Figure 13f shows the geolocation error distribution, which is right-skewed (mean 19.67 km > median 17.54 km), indicating that a small number of higher-error segments pull the mean above the typical performance level. The standard deviation of 8.90 km and the 90th percentile of 32.45 km confirm a relatively compact spread, and the absence of outliers beyond 45 km indicates stable accuracy without catastrophic failures over the observed segment set. The mean geolocation error of 19.67 km over 100 segments is slightly higher than the single-segment result of 17.2 km reported earlier, reflecting the natural variation in channel conditions across the field dataset.

**End-to-end geolocation comparison.** Table 6 reports comprehensive statistics over the 100 segments. The proposed method achieves the lowest mean (19.67 km), tightest dispersion (std 8.90 km), and best worst-case performance (max 44.80 km). TDOA-SDP, Improved-TDOA, and ANN-TDOA yield mean errors of 25.12, 31.42, and 40.23 km, respectively—28%, 60%, and 104% higher. Beyond mean accuracy, the proposed method also exhibits superior robustness: its standard deviation of 8.90 km is the smallest among all methods (versus 10.82–15.61 km for baselines), the 90th-percentile error of 32.45 km is the tightest, and the maximum error of 44.80 km is substantially lower than the 58.74–78.47 km range observed for the other methods. This indicates that the estimator avoids catastrophic failures even under challenging channel conditions. The ranking consistency between TDOA-level (Table 5) and geolocation-level (Table 6) performance confirms that improved delay estimation directly translates to enhanced positioning accuracy through the SDP localization stage.

**CSI ablation on field data.** Table 5 also provides a field-data ablation: removing CSI increases the mean TDOA deviation from 20.3 to 31.7 km. The CSI descriptors comprise signal-derived features (SNR, CFO, spectral characteristics) and IRI-2020 ionospheric parameters ($f_{o}F_{2}$, $h_{mF2}$) obtained from publicly available solar flux and geomagnetic indices [26]. No oracle or ground-truth channel parameters are provided; the observed gain reflects the value of operationally accessible context rather than privileged information. In practical passive monitoring systems where active ionospheric sounding is unavailable, the IRI-2020 model requires only standard inputs (geographic coordinates, UTC time, solar flux F10.7, and Kp index), all of which are routinely available from public archives. The CSI branch therefore enables the estimator to adapt to varying ionospheric conditions without dedicated ionosondes or transmitter cooperation, and the 56% error increase upon removing CSI confirms that even coarse environmental context substantially improves TDOA robustness. The present study did not perform a dedicated sensitivity analysis for perturbations in F10.7 and Kp. Since the IRI-derived ionospheric parameters enter the network only as low-dimensional auxiliary CSI rather than as hard constraints, their uncertainty is expected to affect performance mainly by reducing the benefit of the CSI branch rather than invalidating the full estimator. This interpretation is consistent with the no-CSI ablation on field data, where the estimator remains operational although its accuracy degrades. A more systematic uncertainty-propagation analysis for space-weather input errors will be investigated in future work.

**Limitations.** The dataset covers a single transmitter, session, and frequency; nighttime, storm-time, multi-frequency, and explicit multi-hop conditions remain to be evaluated. The field errors exceed synthetic-data levels at comparable SNR, indicating residual domain shift. The benchmark TDOAs inherit non-negligible uncertainty (±5 km consistency level). Future work will extend validation to broader geographic regions, longer observation periods, and diverse ionospheric activity levels.

**End-to-End Runtime Discussion.** For one localization update under the reference-based formulation, the total latency can be approximated as

(36) $$T_{E2E}\approx T_{\mathrm{TDOA}}+T_{\mathrm{SDP}},$$

where $T_{\mathrm{TDOA}}$ denotes the latency of the M−1 pairwise TDOA inferences and $T_{\mathrm{SDP}}$ denotes the runtime of one SDP solve. In the present six-receiver configuration, five receiver pairs are required for each update. On a workstation equipped with an AMD Ryzen 9 9950X CPU (Advanced Micro Devices, Inc., Santa Clara, CA, USA), a COLORFUL GeForce RTX 5070 Ti GPU (Shenzhen Colorful Yugong Technology and Development Co., Ltd., Shenzhen, China), and 96 GB COLORFUL RAM (Shenzhen Colorful Yugong Technology and Development Co., Ltd., Shenzhen, China), the five pairwise TDOA inferences can be processed in one batch and remain below 20 ms, consistent with the single-forward-pass timing reported in Section 3.2, while one SDP solve requires approximately 0.78 s on average. The resulting end-to-end latency is therefore about 0.8 s per 1.5 s segment, which indicates that segment-level near-real-time processing is feasible on such workstation hardware.

For embedded deployment, using the pruned model reported in Section 3.2 with less than 50 ms per window, the TDOA stage is on the order of 250 ms if the five receiver pairs are processed sequentially. In this case, the localization backend becomes the dominant source of latency. Therefore, further acceleration of the SDP stage would be desirable for stricter real-time requirements on embedded platforms.

## 6. Conclusions

This paper presented an end-to-end framework for HF skywave source geolocation comprising three integrated components. First, an improved wideband HF ionospheric channel model was developed by augmenting the classical ITS structure with IRI-based parameters, regional calibration for the Chinese sector, and a stochastic perturbation module, yielding physics-consistent training and evaluation waveforms. The model’s statistical plausibility was confirmed by comparing its RMS delay spread, Doppler spread, and coherence bandwidth against published HF sounding measurements over mid-latitude paths. Second, a CNN-based TDOA estimator was designed to fuse raw waveform features, multi-weight GCC representations, and optional channel-state information within a unified regression network for robust delay estimation under low-SNR and multipath conditions. Ablation analysis confirmed the necessity of all three branches, with removal of CSI conditioning alone increasing TDOA MAE by 55% on synthetic data and 56% on field recordings. Third, the geolocation problem was formulated as a bias-regularized constrained least-squares model with nonnegative squared-range correction terms and solved via SDP relaxation without requiring prior reflection-height information.

In simulations, the proposed TDOA estimator achieved the lowest MAE across the full −10 to 20 dB SNR range and substantially narrowed the gap to the CRLB at high SNR. End-to-end geolocation via the SDP back end consistently outperformed ANN-TDOA, Improved-TDOA, and TDOA-SDP under identical receiver configurations, with a spatial performance map confirming low error over a broad interior region.

In field experiments with six GPS-synchronized receivers and 100 signal segments from Xi’an, the proposed estimator reduced the mean TDOA deviation by 51% relative to GCC-PHAT (20.3 versus 41.6 km), and the end-to-end pipeline achieved a mean geolocation error of 19.67 km with a 90th-percentile error of 32.45 km, outperforming all baselines. These results were broadly consistent with the simulation findings, supporting the practical applicability of the framework.

Future work will focus on extending the channel model to diverse ionospheric regions, incorporating explicit multi-hop propagation modeling, conducting broader field campaigns across different seasons and solar activity levels, and developing lightweight implementations for real-time deployment in large-scale passive monitoring systems.

## Figures and Tables

**Table 1:** Statistical comparison between the proposed channel model and representative published HF skywave sounding measurements over mid-latitude paths.

Parameter	Proposed Model	Literature Range	References
RMS delay spread (ms)	0.8–2.1	0.5–3.0	[16,17]
Doppler spread (Hz)	0.3–1.8	0.1–2.0	[18,19]
Coherence BW @ 0.5 (kHz)	2.1–4.8	1.5–5.0	[16,17]
$f_{o}F_{2}$ relative variation (%)	∼7	5–10	[16,20]

**Note:** The comparison is statistical rather than realization-specific; the proposed model is configured for the Chinese sector, whereas the literature ranges are drawn from representative mid-latitude HF sounding studies relevant to the studied region.

**Table 2:** Layer configuration and tensor shapes (reproducible setting).

Block	Layer	Ch./Units	Kernel/Lag	Stride	Input → Output
Input	Waveforms $\left(x_{1},x_{2}\right)$	–	*T* = 15,360	–	(2,T)→(2,T)
SincNet	SincConv + BN + ReLU	64	k=101	s=2	$\left(2,\;T\right)\;\to \;\left(64,\;{\tilde{T}}_{1}\right)$
	Conv + BN + ReLU + Pool	64	k=7	s=2	$\left(64,\;{\tilde{T}}_{1}\right)\;\to \;\left(64,\;{\tilde{T}}_{2}\right)$
	Conv + BN + ReLU + GAP	128	k=5	s=2	$\left(64,\;{\tilde{T}}_{2}\right)\;\to \;\left(128\right)$
Multi-GCC	GCC (PHAT, SCOT, ML)	3 maps	D=409	–	(3,409)→(3,409)
	Conv + BN + ReLU	64	k=9	s=2	$\left(3,\;D\right)\;\to \;\left(64,\;{\tilde{D}}_{1}\right)$
	Conv + BN + ReLU + GAP	128	k=7	s=2	$\left(64,\;{\tilde{D}}_{1}\right)\;\to \;\left(128,\;{\tilde{D}}_{2}\right)\;\to \;\left(128\right)$
CSI (opt.)	MLP (ReLU–Drop–ReLU)	$d_{\mathrm{CSI}}\;\to \;32$	–	–	$\left(d_{\mathrm{CSI}}\right)\;\to \;\left(32\right)$
Fusion	Concat(Sinc, GCC, CSI)	–	–	–	128 (Sinc) + 128 (GCC) + [32 if CSI]
Head	FC–ReLU–Drop–FC	288→128→1	–	–	with CSI: (288→128→1). without CSI: (256→128→1)

**Shape convention:** (C,L) denotes *C* channels and length *L* along the 1-D axis (time for waveforms, lag for GCC). **Length propagation:** valid convolution: $L^{\prime }=\left\lfloor \left(L-k\right)/s\right\rfloor +1$; same-padding with stride *s*: $L^{\prime }=\left\lceil L/s\right\rceil$; pooling (2,2): $L^{\prime }=\left\lfloor L/2\right\rfloor$. **Numerical setting:** T=15,360. SincConv (k=101, s=2, valid), Conv (k=7, s=2, same) + Pool (2,2), Conv (k=5, s=2, same): ${\tilde{T}}_{1}=7630$, ${\tilde{T}}_{2}=1907$, pre-GAP length 954. GCC: D=409; $\left(k=9,\;s=2\right)\;\Rightarrow \;{\tilde{D}}_{1}=205$; $\left(k=7,\;s=2\right)\;\Rightarrow \;{\tilde{D}}_{2}=103$. **Waveform representation:** Each receiver contributes one complex-baseband stream reconstructed from recorded I/Q samples. **Output convention:** Single-pair regressor; in multi-receiver operation, reference-based TDOAs are obtained by repeated evaluation.

**Table 3:** Ablation study on synthetic test data.

Variant	TDOA MAE (μs)	Acc@30 km (%)
Full (Waveform + GCC + CSI)	10.2	68.5
w/o CSI	15.8	56.3
Waveform-only (SincNet)	18.4	51.7
GCC-only (multi-weight)	24.6	44.2

**Table 4:** Single-segment TDOA estimates and absolute deviations from the benchmark reference (km).

	Reference	GCC-PHAT		FS-GCC		MATE		PROPOSED		PROPOSED (No CSI)	
Receiver	TDOA	TDOA	Error	TDOA	Error	TDOA	Error	TDOA	Error	TDOA	Error
Chengdu	283.4	260.5	22.9	245.8	37.6	251.2	32.2	300.9	17.5	311.5	28.1
Shenzhen	−560.1	−598.6	38.5	−592.4	32.3	−593.8	33.7	−580.2	20.1	−590.5	30.4
Shanghai	−312.8	−351.4	38.6	−347.5	34.7	−344.2	31.4	−332.1	19.3	−342.4	29.6
Wuyishan	−226.5	−265.8	39.3	−260.1	33.6	−254.3	27.8	−242.3	15.8	−251.8	25.3
Kunming	−304.9	−343.2	38.3	−338.6	33.7	−335.1	30.2	−323.5	18.6	−331.8	26.9
Mean Error	–	–	35.5	–	34.4	–	31.1	–	18.3	–	28.1

**Table 5:** Mean absolute deviation from the benchmark TDOA reference over 100 field segments (km).

Receiver	GCC-PHAT	FS-GCC	MATE	PROPOSED	PROPOSED (No CSI)
Chengdu	25.5	40.4	36.4	19.8	31.4
Shenzhen	48.2	42.0	38.5	22.2	35.7
Shanghai	46.3	40.2	35.8	21.7	33.5
Wuyishan	43.4	36.8	30.5	17.4	28.2
Kunming	44.8	38.5	33.0	20.3	29.6
Mean	41.6	f 39.6	34.8	20.3	31.7

**Table 6:** End-to-end geolocation statistics over 100 field segments (km).

Method	Mean	Std	Median	90th %ile	Max
ANN-TDOA	40.23	15.61	37.85	62.12	78.47
Improved-TDOA	31.42	12.31	29.14	48.76	65.23
TDOA-SDP	25.12	10.82	23.69	41.25	58.74
PROPOSED	19.67	8.90	17.54	32.45	44.80

## Data Availability

The data presented in this study are available from the corresponding author upon reasonable request. The data are not publicly released because they are subject to project-related access restrictions and measurement-campaign data management requirements.

## References

[1] Zhang L., Wang D., Wu Y. (2014). Performance analysis of TDOA and FDOA location by differential calibration with calibration sources. J. Commun..

[2] Xu C., Wang Z., Wang Y., Wang Z., Yu L. (2020). Three passive TDOA-AOA receivers-based flying-UAV positioning in extreme environments. IEEE Sens. J..

[3] Karmy M., ElSayed S., Zekry A. (2020). Performance enhancement of an indoor localization system based on visible light communication using RSSI/TDOA hybrid technique. J. Commun..

[4] Ghadiri-Modarres M., Mojiri M., Karimi-Ghartemani M. (2015). New adaptive algorithm for delay estimation of sinusoidal signals with unknown frequency. IEEE Trans. Instrum. Meas..

[5] Pallotta L., Giunta G. (2022). Accurate delay estimation for multisensor passive locating systems exploiting the cross-correlation between signals cross-correlations. IEEE Trans. Aerosp. Electron. Syst..

[6] Knapp C., Carter G. (1976). The generalized correlation method for estimation of time delay. IEEE Trans. Acoust. Speech Signal Process..

[7] Garcia-Barrios G., Gutierrez-Arriola J.M., Saenz-Lechon N., Osma-Ruiz V.J., Fraile R. (2021). Analytical model for the relation between signal bandwidth and spatial resolution in steered-response power phase transform (SRP-PHAT) maps. IEEE Access.

[8] Diaz-Guerra D., Miguel A., Beltran J.R. (2021). Robust sound source tracking using SRP-PHAT and 3D convolutional neural networks. IEEE/ACM Trans. Audio Speech Lang. Process..

[9] Kirmaz A., Şahin T., Michalopoulos D.S., Gerstacker W. (2023). ToA and TDoA estimation using artificial neural networks for high-accuracy ranging. IEEE J. Sel. Areas Commun..

[10] Xu C., Cai H., Gao S., Yan X. (2023). A method for HF skywave source geolocation in unknown ionosphere environments and experimental results. IEEE Antennas Wirel. Propag. Lett..

[11] Xia N., Xing B. (2021). A direct localization method for HF source geolocation and experimental results. IEEE Antennas Wirel. Propag. Lett..

[12] Xiong W., Schindelhauer C., So H.C. (2023). Globally optimized TDOA high-frequency source localization based on quasi-parabolic ionosphere modeling and collaborative gradient projection. IEEE Trans. Aerosp. Electron. Syst..

[13] Yang G., Sun L., Wei W., Zhang L. (2021). A novel land-based high-frequency geolocation system. IEEE Geosci. Remote Sens. Lett..

[14] Xu C., Cai H., Gao S., Zhai Q. (2023). An improved TDOA method for land-based long-range HF skywave source geolocation and experimental results. Sensors.

[15] Vogler L.E., Hoffmeyer J.A. (1993). A model for wideband HF propagation channels. Radio Sci..

[16] Angling M.J., Cannon P.S., Davies N.C., Willink T.J., Jodalen V., Lundborg B. (1998). Measurements of Doppler and multipath spread on oblique high-latitude HF paths and their use in characterizing data modem performance. Radio Sci..

[17] Xue R., Wang Y. An improved high-latitude HF channel model based on ray tracing with time-varying parameter factor. Proceedings of the 8th IEEE International Conference on Computer and Communications (ICCC).

[18] Davies K. (1990). Ionospheric Radio.

[19] Yu X., Lu A.-A., Gao X., Li G.Y., Ding G., Wang C.-X. (2022). HF skywave massive MIMO communication. IEEE Trans. Wirel. Commun..

[20] Bilitza D., Altadill D., Truhlik V., Shubin V., Galkin I., Reinisch B., Huang X. (2017). International Reference Ionosphere 2016: From ionospheric climate to real-time weather predictions. Space Weather.

[21] Yan Z., Wang G., Han Y., Che M.M., Su D.L., Rahman T. The modification of IRI model for Chinese region and its verification. Proceedings of the 4th IEEE International Symposium on Microwave, Antenna, Propagation and EMC Technologies for Wireless Communications (MAPE).

[22] Wu J., Quan K.H., Dai K.L., Luo F.G., Sun X.R., Li Z.Q., Cao C., Liu R.Y., Shen C.S. (1996). Progress in the study of the Chinese Reference Ionosphere. Adv. Space Res..

[23] Wang J., Feng F., Bai H.M., Cao Y.B., Chen Q., Ma J.G. (2020). A regional model for the prediction of M(3000)F2 over East Asia. Adv. Space Res..

[24] Yu X., Gao X., Lu A.-A., Zhang J., Wu H., Li G.Y. (2023). Robust precoding for HF skywave massive MIMO. IEEE Trans. Wirel. Commun..

[25] Ravanelli M., Bengio Y. Speaker recognition from raw waveform with SincNet. Proceedings of the IEEE Spoken Language Technology Workshop (SLT).

[26] Bilitza D., Pezzopane M., Truhlik V., Altadill D., Reinisch B.W., Pignalberi A. (2022). International Reference Ionosphere 2020: Model description and major changes. J. Geophys. Res. Space Phys..

[27] Cobos M., Antonacci F., Comanducci L., Sarti A. (2020). Frequency-sliding generalized cross-correlation: A sub-band time delay estimation approach. IEEE/ACM Trans. Audio Speech Lang. Process..

[28] Ji Q., Wu H. (2025). Adaptive modulation tracking for high-precision time-delay estimation in multipath HF channels. Sensors.

